# The (r)evolution of cancer genetics

**DOI:** 10.1186/1741-7007-8-74

**Published:** 2010-06-11

**Authors:** Francesca D Ciccarelli

**Affiliations:** 1 Department of Experimental Oncology, European Institute of Oncology, IFOM-IEO Campus, Via Adamello 16, 20139 Milan, Italy

## Abstract

The identification of an increasing number of cancer genes is opening up unexpected scenarios in cancer genetics. When analyzed for their systemic properties, these genes show a general fragility towards perturbation. A recent paper published in *BMC Biology *shows how the founder domains of known cancer genes emerged at two macroevolutionary transitions - the advent of the first cell and the transition to metazoan multicellularity.

See research article http://www.biomedcentral.com/1741-7007/8/66

## Increasing number and heterogeneity of cancer genes

Recent advances in sequencing technologies and the launching of massive resequencing projects such as the Cancer Genome Project [1] have boosted the production of cancer genomics data. In the past few years, the entire repertoire of human exons has been sequenced in glioblastoma [2], pancreatic [3], breast and colorectal [4] cancers, and somatic mutations in selected genes have been mapped in multiple samples of renal [5] and lung [6] adenocarcinomas. In addition, the whole genomes of individuals affected by leukemia [7,8], melanoma [9], glioma [10], breast [11,12], and lung [13] cancers have been fully resequenced. All these studies have led to the identification of more than 1,000 potential cancer genes, and the list is likely to grow in the near future.

**Figure 1:**
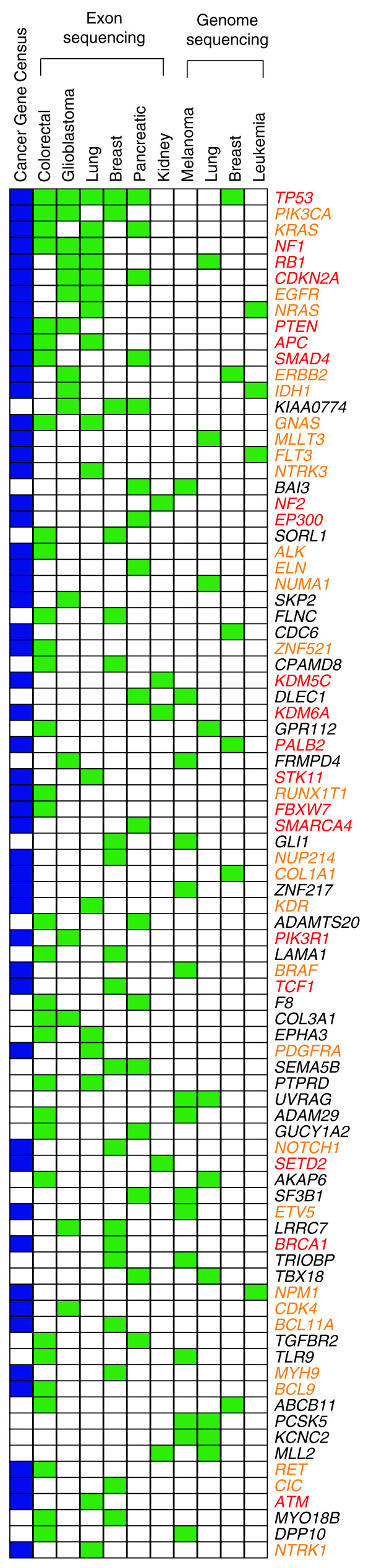
**Heterogeneity of genes mutated in different cancer types**. So far, more than 1,000 human genes have been identified that carry proven or potential driver mutations involved in cancer progression. Of those, only 85 genes have been found mutated in at least two studies, either in large-scale screenings or in the cancer gene census, which is a literature-based collection of known cancer genes [20]. The latter can be genetically repressive (red) or dominant (orange), which broadly correspond to tumor-suppressors and caretakers and to oncogenes, respectively. This distinction cannot be done for genes identified through large-scale screenings that involve either massive exon [2-6] or whole-genome [7-13] resequencing.

This massive amount of information will have a huge impact on our understanding of cancer genetics, even more so considering that the biological role of most mutations is still obscure. These first unbiased screenings have led to the identification of novel and unsuspected determinants of cancer, such as the isocitrate dehydrogenase enzyme genes *IDH1 *and *IDH2*, which have been found mutated in glioblastoma multiforme [2]. They have also started to question some cornerstones of cancer biology, such as the description of cancer as a unique disease driven by the somatic modification of a few key regulators. The progressive identification of novel mutated genes is expanding the 'cast of actors' [14] whose mutations might be causally involved in driving cancer. Moreover, given the high heterogeneity of genes mutated in different cancer types (Figure 1), the overall 'plot' is becoming more intricate. The emerging picture suggests that there may be distinct genetic routes to reach the common aftermath of all tumorigenic processes, which is uncontrolled cell proliferation. For example, as many as 12 core pathways are disrupted in the majority of pancreatic cancers through multiple somatic mutations [3]. This opens up an intriguing scenario where the deregulation of key pathways for tumorigenesis represents only the final step of a more general perturbation of cellular activity. The cell is seen as an integrated system in which all processes form a tightly interconnected network more than as an ensemble of independent pathways. In this context, the effect of somatic mutations occurring in the cancer genome should be interpreted in the light of their broader impact on the system's equilibrium.

Cancer is a disease of multicellular organisms, where each cell is integrated within the larger system of the whole organism. In a recent paper in *BMC Biology*, Domazet-Lošo and Tautz [15] trace the evolutionary origins of known cancer genes and find that in most cases the origins coincide with one of two pivotal events in evolution - the emergence of the first cell or the transition towards metazoan multicellularity.

## Systems-level perturbations of cancer-related mutations

The first attempts to study cancer genes using more systematic approaches have already proved successful in broadening our knowledge of the genetic determinants of cancer. A multidimensional analysis has combined sequence similarity, functional annotations, protein-protein interactions, and molecular pathways to examine genes mutated in breast and colorectal cancers [16]. This study showed that while processes involved with intracellular signaling, control of the cell cycle and metabolism are modified in both tumors, most pathways are instead specific to one of the two, suggesting that different molecular mechanisms underlie these two types of tumors [16].

In the context of systems-biology approaches, methods that examine protein-protein interactions are particularly informative because they evaluate the effect of mutations on the complex network of cellular interconnections. Topological analyses of the human protein-protein interaction network reveal that known and candidate cancer genes encode central hubs - that is, proteins that engage several connections and occupy central positions at the crossroads of multiple biological processes [17,18]. Our analysis [18] also reveals that paralogs of cancer genes (that is, genes related by duplication) are less common than for other genes, which indicates a sensitivity of cancer genes to dosage modification.

All these properties are uncommon within the human gene repertoire and help to interpret the incidence of somatic mutations as a sign of a broader fragility of cancer genes towards any type of perturbation. Deletions, mutations and amplifications of highly interconnected genes are likely to be deleterious because they can affect several aspects of the cell's life. Interestingly, we have found that these properties are not limited to well-known cancer genes but are also shared by genes whose modifications have been identified through large-scale mutational screenings [19]. Again, this shows that there are common features of cancer genes, not immediately apparent from their individual function, but seen at a systems level, that help explain their role in tumor development.

## Evolutionary origin of cancer genes

The newest piece of evidence that confirms how useful the global analysis of cancer genes can be is reported by Domazet-Lošo and Tautz [15], who analyze the origin of founder domains of known cancer proteins. Using a methodology called 'genomic phylostratigraphy', the authors are able to trace when the most conserved portions of known cancer proteins, which often correspond to functional domains, appeared in evolution. They observe two peaks, one at the origin of the ancestral cell and the other at the origin of metazoans.

Interestingly, these two peaks are enriched in two distinct groups of genes, namely 'caretakers' and 'gatekeepers', that contribute to cancer through different mechanisms. Caretakers are associated with the origin of the first cell and are involved in the control of genome stability and their modification increases the mutation rate and favors genomic instability. Gatekeepers originated with metazoans and their modifications directly or indirectly affect cell differentiation, growth and death. The different evolutionary origins of these genes suggest two distinct mechanisms of carcinogenesis. The first deals with the basic functions of the cell, such as the control of genome stability, that, very reasonably, were established already in the ancestral eukaryotic cell. The second mechanism is intimately connected to multicellularity and to the interactions between cells within a complex organism. This observation puts cancer in the context of macroevolutionary transitions and links tumorigenesis to the disruption of processes that are essential for survival of the cell and for its communication with the external environment.

The emerging heterogeneity of the cancer genomic landscape has been used to question the usefulness of large-scale screenings, the main concern being that the discovery of rare mutations adds very little to the overall knowledge of cancer genetics. Approaches that focus on the identification of global features, more than to the study of single genes, show instead that a comprehensive catalogue of cancer genetic determinants is instrumental to trace recurrent patterns in their systems-level and evolutionary properties.
